# Supplementary material for: The Relationship between Emotion Regulation (ER) and Problematic Smartphone Use (PSU): A Systematic Review and Meta-Analyses
Source: Int J Environ Res Public Health. 2022 Nov 28;19(23):15848. doi: 10.3390/ijerph192315848 (PMC9740505; doi:10.3390/ijerph192315848)
Supplement: Supplementary file 1 [file ijerph-19-15848-s001.zip › ijerph-1913237-supplementary.pdf]

Table S1. Quality assessment of included studies.

Study	NHMRC level of evidence & Study Design	1	2	3	4	5	6	7	8	9	10	11	Total
Amendola et al., 2018 [59]	IV: cross-sectional	Y	Y	Y	N	Y	Y	Y	Y	Y	N	Y	9/11 (82%)
Chen et al., 2017 [60]	IV: cross-sectional	Y	Y	Y	N	Y	Y	Y	Y	Y	N	Y	9/11 (82%)
Coco et al., 2020 [51]	IV: cross-sectional	Y	Y	Y	N	Y	Y	Y	Y	Y	Y	Y	10/11 (91%)
Domoff et al., 2020 [61]	IV: cross-sectional	Y	Y	Y	N	Y	Y	Y	Y	Y	N	Y	9/11 (82%)
Elhai et al., 2016 [40]	IV: cross-sectional	Y	Y	Y	Y	Y	Y	Y	Y	Y	Y	Y	11/11 (100%)
Elhai and Contractor, 2018 [56]	IV: cross-sectional	Y	Y	Y	N	Y	Y	Y	Y	Y	Y	Y	10/11 (91%)
Elhai et al., 2018 [57]	IV: cross-sectional	Y	Y	Y	N	Y	Y	Y	Y	Y	Y	Y	10/11 (91%)
Ercengiz et al., 2020 [62]	IV: cross-sectional	Y	Y	Y	N	Y	Y	Y	Y	Y	N	Y	9/11 (82%)
Extremera et al., 2019 [63]	IV: cross-sectional	Y	Y	Y	N	Y	Y	Y	Y	Y	N	Y	9/11 (82%)
Fortes et al., 2021 [64]	IV: cross-sectional	Y	Y	Y	N	Y	Y	Y	Y	Y	N	Y	9/11 (82%)
Fu et al., 2020 [65]	IV: cross-sectional	Y	Y	Y	N	Y	Y	Y	Y	Y	N	Y	9/11 (82%)
Giordano et al., 2021 [49]	IV: cross-sectional	Y	Y	Y	N	Y	Y	Y	Y	Y	Y	Y	10/11 (91%)
Hoffner and Lee, 2015 [68]	IV: cross-sectional	Y	Y	Y	N	Y	N	Y	Y	Y	N	Y	8/11 (73%)
Horwood and Anglim, 2021 [58]	IV: cross-sectional	Y	Y	Y	N	Y	Y	Y	Y	Y	Y	Y	10/11 (91%)
lo Coco et al., 2020 [50]	IV: cross-sectional	Y	Y	Y	N	Y	Y	Y	Y	Y	Y	Y	10/11 (91%)
Rozgonjuk and Elhai, 2021 [52]	IV: cross-sectional	Y	Y	Y	N	Y	Y	Y	Y	Y	N	Y	9/11 (82%)

Table S1. Cont.

Study	NHMRC level of evidence & Study Design	1	2	3	4	5	6	7	8	9	10	11	Total
Sakiroglu, 2019 [53]	IV: cross-sectional	Y	Y	N	N	Y	Y	Y	Y	Y	N	Y	8/11 (73%)
Satici and Deniz, 2020 [47]	IV: cross-sectional	Y	Y	Y	N	Y	Y	Y	Y	Y	N	Y	9/11 (82%)
Squires et al., 2020 [48]	IV: cross-sectional	Y	Y	Y	N	Y	Y	Y	Y	Y	N	Y	9/11 (82%)
Yildiz et al., 2017 [66]	IV: cross-sectional	Y	Y	Y	N	Y	Y	Y	Y	Y	N	Y	9/11 (82%)
Zsido et al., 2021 [67]	IV: cross-sectional	Y	Y	Y	N	Y	Y	Y	Y	Y	N	Y	9/11 (82%)

Meta-analysis: correlation

Variable for studies	Study
Variable for number of cases	N
Variable for correlation coefficients	r

Study	Sample size	Correlation coefficient	95% CI	z	P	Weight (%)	
						Fixed	Random
Giordano 2021-A	252	0.438	0.332 to 0.533			6.63	8.26
Fu 2019	720	0.440	0.379 to 0.497			19.08	8.67
lo Coco 2020-B	280	0.393	0.289 to 0.488			7.37	8.32
Coco 2020	242	0.392	0.280 to 0.494			6.36	8.23
lo Coco 2020-G	367	0.427	0.339 to 0.507			9.69	8.45
Rozgonjuk 2019	300	0.156	0.0435 to 0.265			7.91	8.35
Squires 2020	204	0.362	0.236 to 0.476			5.35	8.11
Satici 2020	320	-0.300	-0.397 to -0.197			8.44	8.39
Elhai 2016	308	0.130	0.0185 to 0.238			8.12	8.37
Giordano 2021-M	252	0.246	0.126 to 0.359			6.63	8.26
Giordano 2021-F	252	0.251	0.132 to 0.363			6.63	8.26
Sakiroglu 2019	296	0.410	0.311 to 0.501			7.80	8.35
Total (fixed effects)	3793	0.298	0.269 to 0.327	18.833	<0.001	100.00	100.00
Total (random effects)	3793	0.286	0.160 to 0.402	4.360	<0.001	100.00	100.00

Test for heterogeneity

Q	184.2011
DF	11
Significance level	$P < 0.0001$
I^2 (inconsistency)	94.03%
95% CI for I^2	91.28 to 95.91

(a)

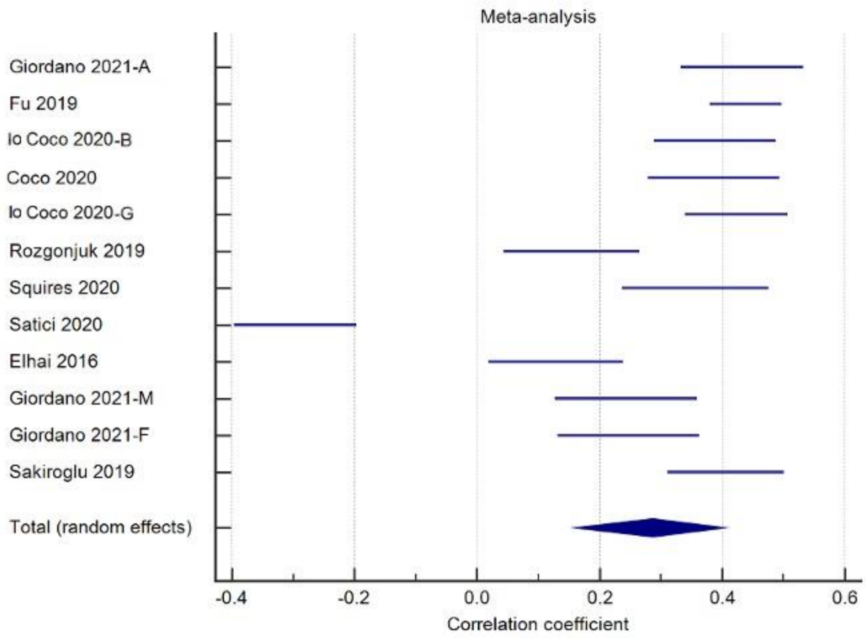

(b)

Figure S1: **(a)** Meta-analysis calculating the pooled correlation coefficient of emotional dysregulation with PSU ; **(b)** forest plot showing the relationship between emotional dysregulation and PSU.
